# Author Correction: In situ visualization of endothelial cell-derived extracellular vesicle formation in steady state and malignant conditions

**DOI:** 10.1038/s41467-025-64770-8

**Published:** 2025-10-03

**Authors:** Georgia K. Atkin-Smith, Jascinta P. Santavanond, Amanda Light, Joel S. Rimes, Andre L. Samson, Jeremy Er, Joy Liu, Darryl N. Johnson, Mélanie Le Page, Pradeep Rajasekhar, Raymond K. H. Yip, Niall D. Geoghegan, Kelly L. Rogers, Catherine Chang, Vanessa L. Bryant, Mai Margetts, M. Cristina Keightley, Trevor J. Kilpatrick, Michele D. Binder, Sharon Tran, Erinna F. Lee, Walter D. Fairlie, Dilara C. Ozkocak, Andrew H. Wei, Edwin D. Hawkins, Ivan K. H. Poon

**Affiliations:** 1 Walter and Eliza Hall Institute of Medical Research, Melbourne, VIC Australia; 2 Department of Medical Biology, The University of Melbourne, Melbourne, VIC Australia; 3 Department of Biochemistry and Chemistry, School of Agriculture, Biomedicine and Environment, La Trobe Institute for Molecular Science, La Trobe University, Melbourne, VIC Australia; 4 Research Centre for Extracellular Vesicles, La Trobe University, Melbourne, VIC Australia; 5 Clinical Haematology Department, Peter MacCallum Cancer Centre and Royal Melbourne Hospital, Melbourne, VIC Australia; 6 Materials Characterisation and Fabrication Platform, Department of Chemical Engineering, University of Melbourne, Parkville, VIC Australia; 7 ARAFlowCore, Alfred Research Alliance, Monash University, Melbourne, VIC Australia; 8 Department of Clinical Immunology and Allergy, Royal Melbourne Hospital, Melbourne, VIC Australia; 9 Department of Rural Clinical Sciences, La Trobe Rural Health School, Bendigo, VIC Australia; 10 Florey Institute of Neuroscience and Mental Health, Melbourne, VIC Australia; 11 Florey Department of Neuroscience and Mental Health, University of Melbourne, Melbourne, VIC Australia; 12 Olivia Newton-John Cancer Research Institute, Heidelberg, VIC Australia; 13 School of Cancer Medicine, La Trobe University, Bundoora, VIC Australia

**Keywords:** Cellular imaging, Cell biology, Exocytosis

Correction to: *Nature Communications* 10.1038/s41467-024-52867-5, published online 22 October 2024

In the version of the article initially published, the flow cytometry shown in the second panel of Fig. 6a for “E*μ*Myc” was an inadvertent duplicate of the first panel “Steady state.” The legend for Fig. 6c has been amended to include the text “Steady state control data also shown in Fig. 4a.” The figure and legend are amended in the HTML and PDF versions of the article.

