# Supplementary material for: In situ visualization of endothelial cell-derived extracellular vesicle formation in steady state and malignant conditions
Source: Nat Commun. 2024 Oct 22;15:8802. doi: 10.1038/s41467-024-52867-5 (PMC11496675; doi:10.1038/s41467-024-52867-5)
Supplement: Supplementary file 1 — Supplementary Information [file 41467_2024_52867_MOESM1_ESM.pdf]

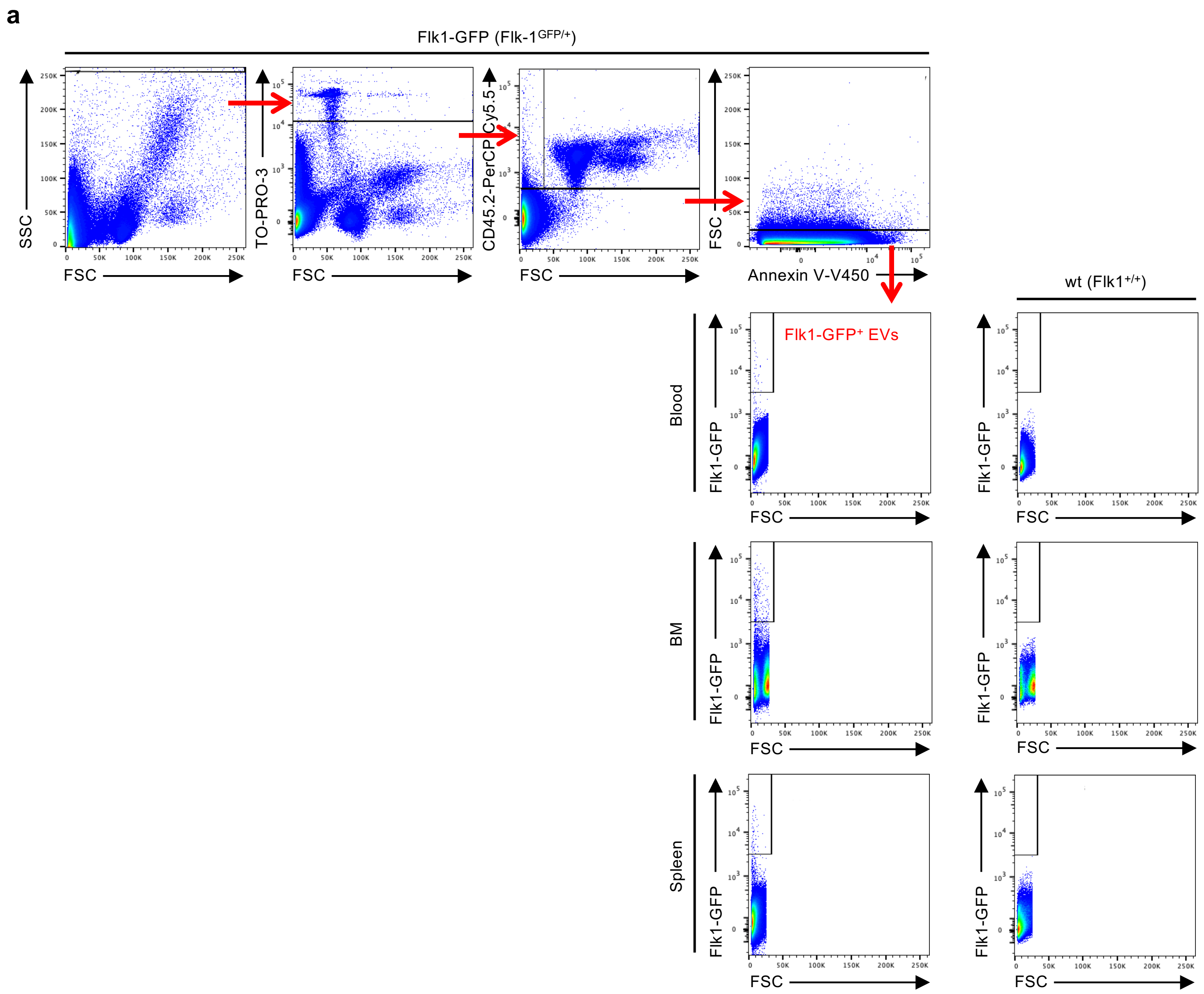

Supplementary Figure 1: Flk1-GFP+ EV gating strategy.

To identify Flk1-GFP+ EVs from tissue samples, events were first separated from SSC high beads; Non permeabilized cells (TO-PRO-3 high) were removed; CD45.2 low events were next selected; FSC low, small particles with low to high Annexin V staining were then selected to exclude FSC high cells; GFP high events were then selected. GFP gates were set based the background GFP fluorescence present in wt control tissue samples. Positive and negative populations were identified based on unstained controls used.

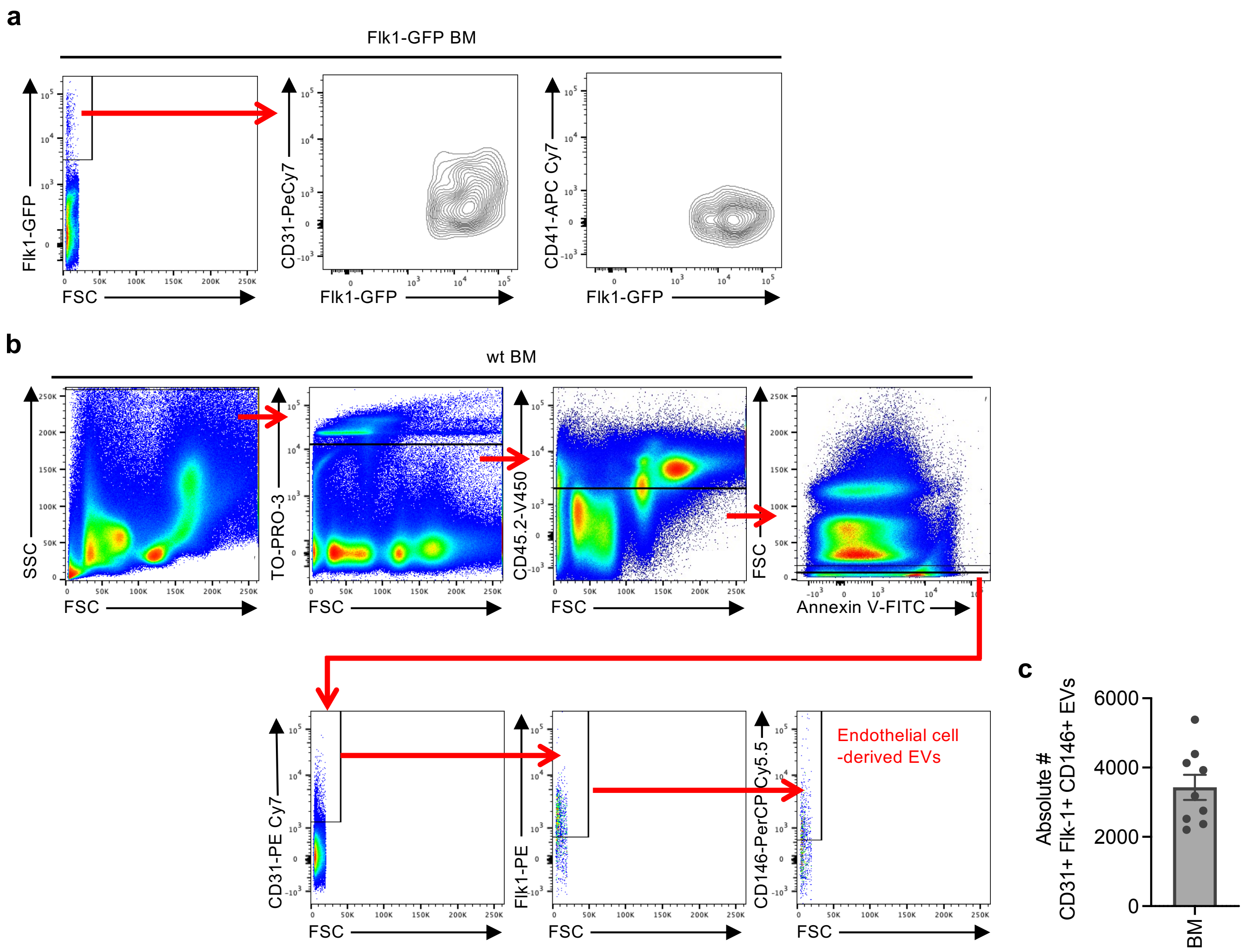

Supplementary Figure 2: Endothelial cell-derived EVs possess common endothelial cell markers.
a Flow cytometry plots showing the level of CD31 and CD41 on BM-derived Flk1-GFP^{+} EVs. Two independent experiments, n=5. **b** Gating strategy used to identify endothelial cell-derived EVs in wt mice with no Flk1-GFP reporter. Events were first separated from SSC high beads; Non permeabilized cells (TO-PRO-3 high) were removed; CD45.2 low events were selected; FSC low, small particles with low to high Annexin V staining were selected to remove FSC high cells; CD31^{+}, Flk1^{+} and CD146^{+} endothelial cell-derived EVs were subsequently gated. Positive and negative populations were identified based on unstained controls used. **c** Flow cytometry quantification of endothelial cell-derived EVs found in wt mouse BM. Three independent experiments, n=9, where data points represent individual mice.

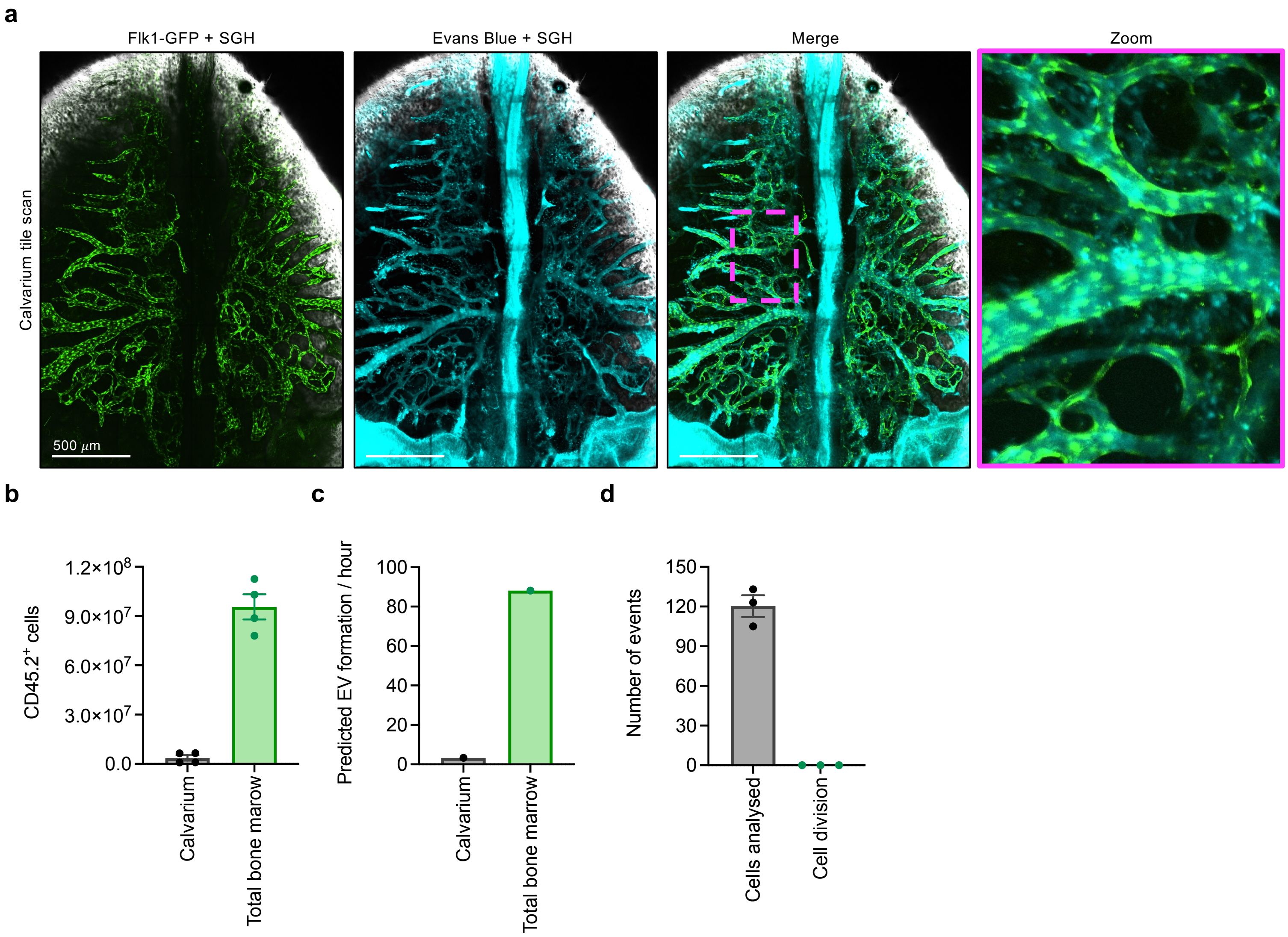

Supplementary Figure 3: Flk1-GFP$^+$ endothelial cell and EV biology.
a. Intravital microscopy tile scan of Flk1-GFP mice intravenously administered with the vascular dye Evans Blue to label the blood vessel network. Data presented as a maximum intensity projection and representative of n=4 independent repeats. **b** Absolute numbers of CD45.2$^+$ immune cells in the calvarium, compared to a 'total bone marrow' including including tibias, femurs, hips, humeri, sternum and calvarium. Total of n=4 mice, data points represent individual mice. **c** Predicted EV formation in the calvarium and total bone marrow was extrapolated from data shown in Main Figure 1j and Supplementary Fig 3b. EV formation in the calvarium was first determined by multiply EV formation in the calvarium imaging area by the imaging area / total calvarium area. Next, predicted EV formation total bone marrow was determined by multiplying the predicted EV formation in the calvarium by the percentage of CD45.2$^+$ immune cells in the calvarium / CD45.2$^+$ in total bone marrow. **d** Flk1-GFP$^+$ endothelial cell division was quantified by tracking Flk1-GFP$^+$ cells during 4D time-lapse experiments of the calvarium bone marrow (average 3D area of 892378748 μm^3, imaged for a total of 4-8 hours. Data represents total number of cells tracked and cell division events observed in three independent repeats.

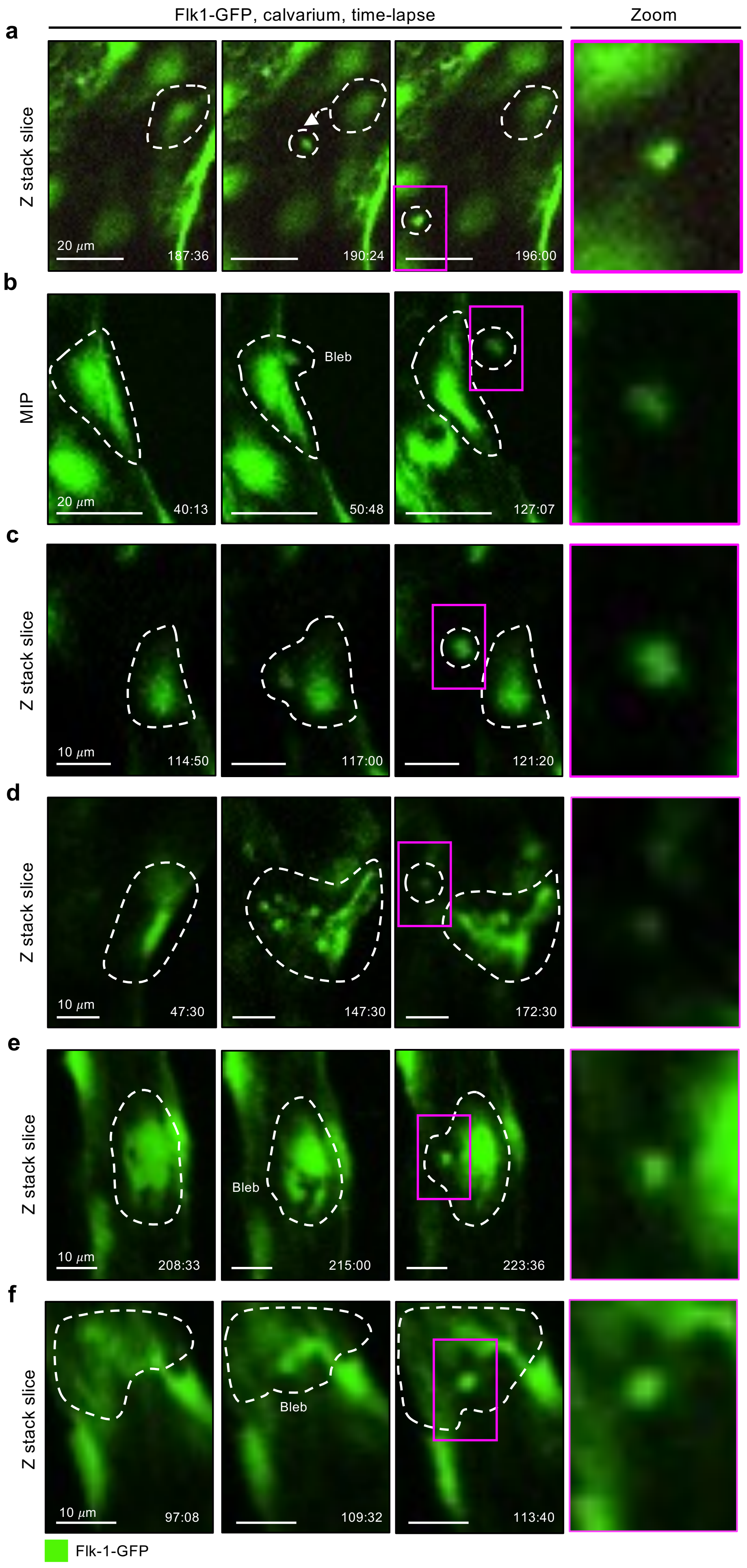

Supplementary Figure 4: Examples of *in vivo* Flk1-GFP⁺ EV formation.
Intravital microscopy of the BM calvarium of Flk1-GFP mice showing the *in situ* formation of Flk1-GFP⁺ EVs. Examples shown represent either an individual 5 μm z slice (a, c, d, e, f) or a maximum intensity projection (MIP, b). Time stamp represents the time post-commencement of imaging.

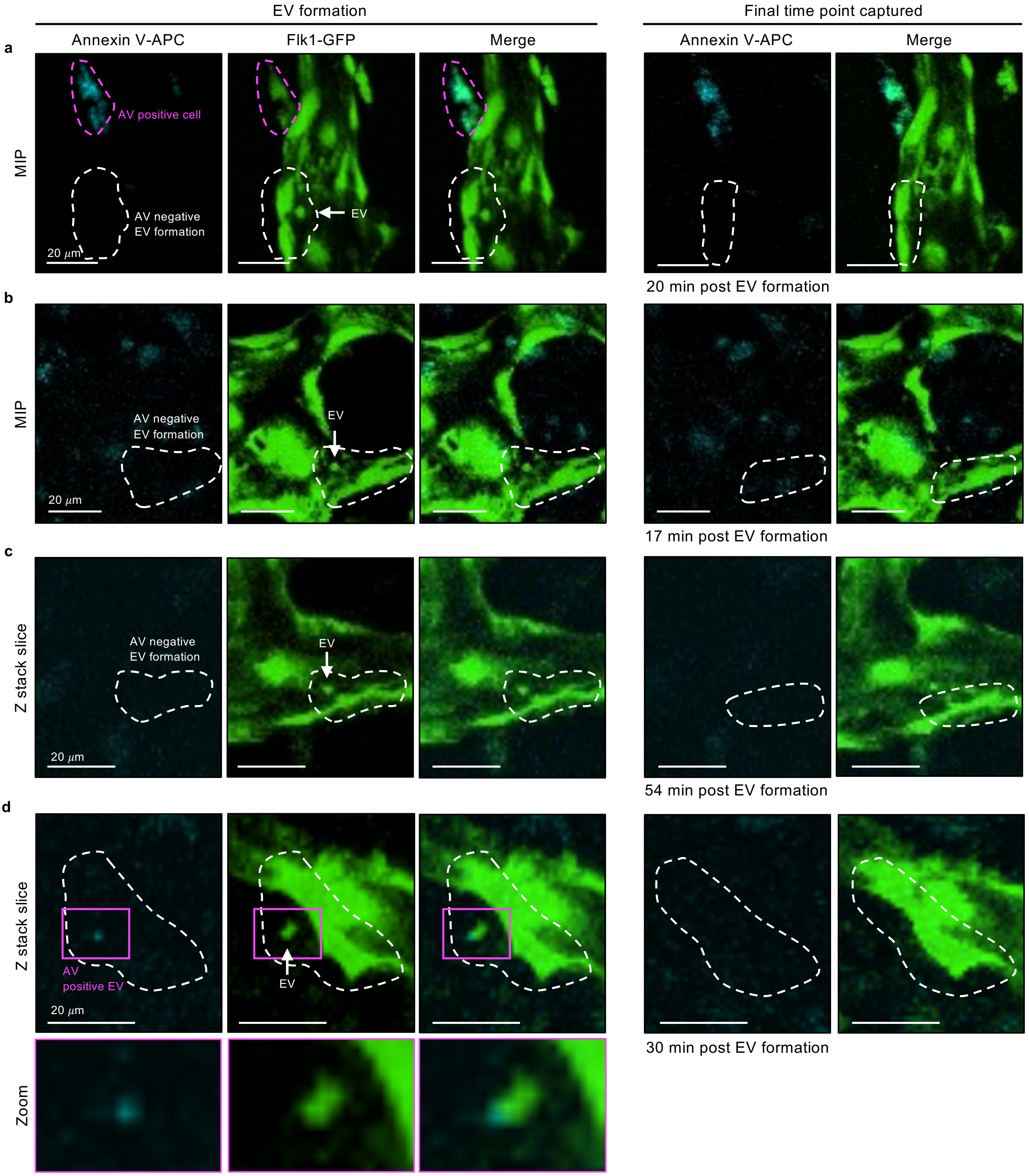

Supplementary Figure 5: Time-lapse intravital microscopy examining Annexin V positive cells and EV formation.
Intravital microscopy of the BM calvarium of Flk1-GFP mice, intravenously injected with ~100 μL Annexin V (AV)-APC. Examples of Flk1-GFP$^+$ EV formation shown represent either a maximum intensity projection (MIP, a, b) or an individual 5 μm z slice (c, d). The right-hand side panels show the last time point captured in the imaging experiment. Data representative of three independent repeats.

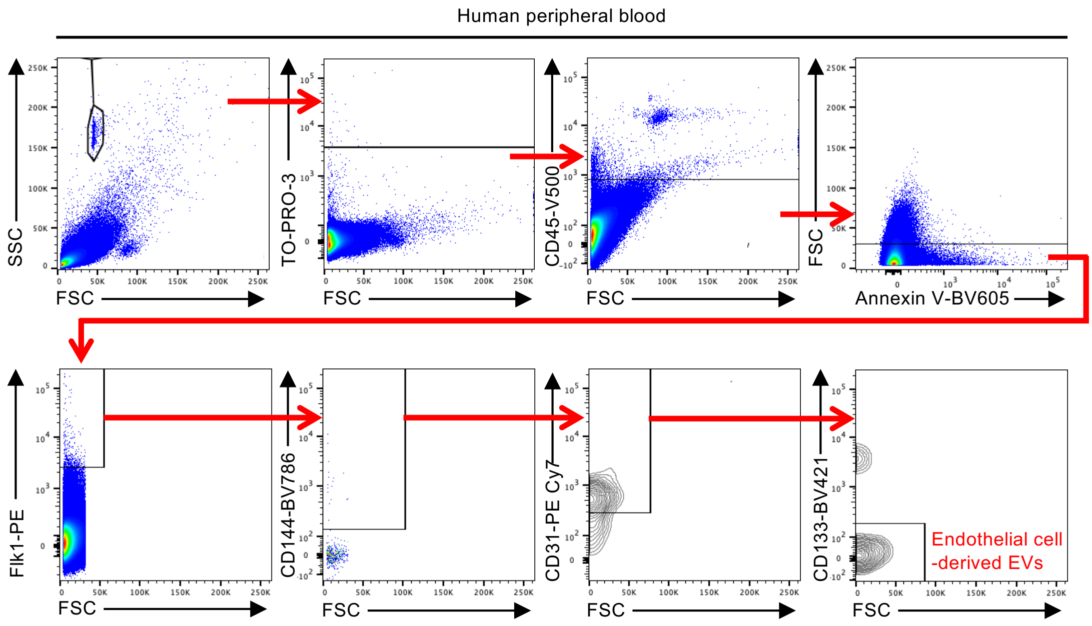

Supplementary Figure 6: Gating strategy to identify human endothelial cell-derived EVs.
To identify endothelial cell-derived EVs from human peripheral blood by flow cytometry, enriched samples were gated as follows: Counting beads were removed; TO-PRO-3 high particles were removed; CD45 low particles were selected; FSC low, small particles with low to high Annexin V staining were selected; Endothelial cell markers including Flk1$^+$, CD144$^+$ and CD31$^+$ events were subsequentially selected; Remaining hematopoietic/stem cell CD133$^+$ particles were removed. Positive and negative populations were identified based on unstained controls used.

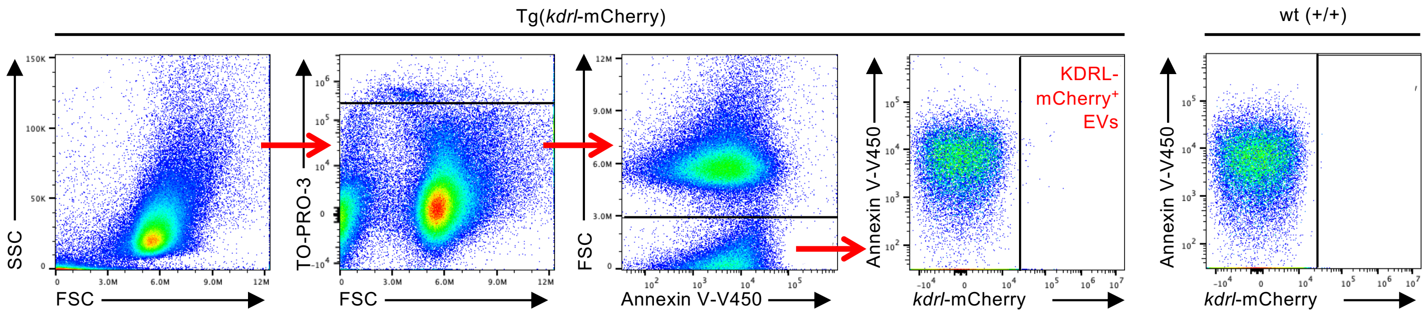

Supplementary Figure 7: KDRL-mCherry zebrafish-derived EV gating strategy.
To identify *kdrl*-mCherry$^+$ EVs in dissociated zebrafish embryos, events were first removed from SSC high beads; Non permeabilized cells (TO-PRO-3 high) were next removed; FSC low, small particles with low to high Annexin V staining were selected and gated on for high mCherry fluorescence. mCherry gate was set based on non-reporter wt embryos. Positive and negative populations were identified based on unstained controls used.

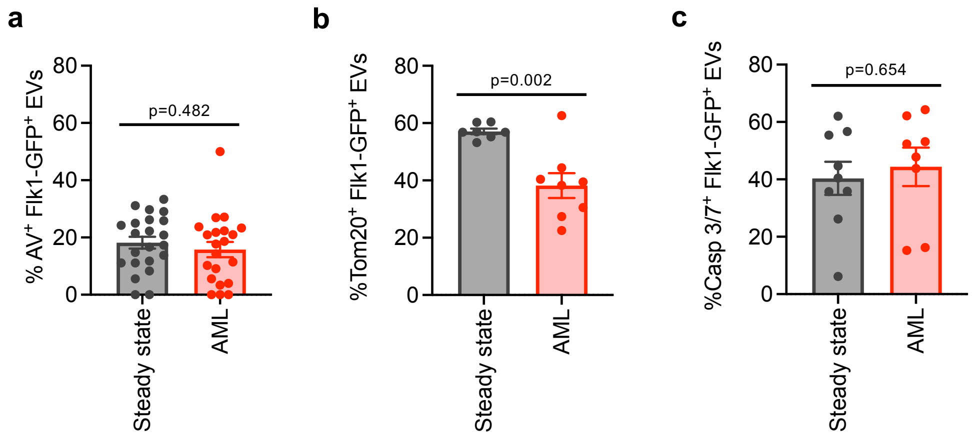

Supplementary Figure 8: Characteristics of Flk1-GFP$^+$ EVs generated during AML.
Flow cytometry comparison of the proportion of AV$^+$ (**a,** n=21-22), Tom20$^+$ (**b,** n=7-8) and cleaved caspase 3/7$^+$ (**c,** n=8-9) Flk1-GFP$^+$ EVs between untreated and AML-burdened mice at day 18 post transplantation. **a** represents blood-derived EVs, **b** and **c** represent BM-derived EVs. Flow cytometry analysis includes merge data of at least three independent experiments where data points represent individual mice. Statistical significance was determined by unpaired Students two tailed *t* test.

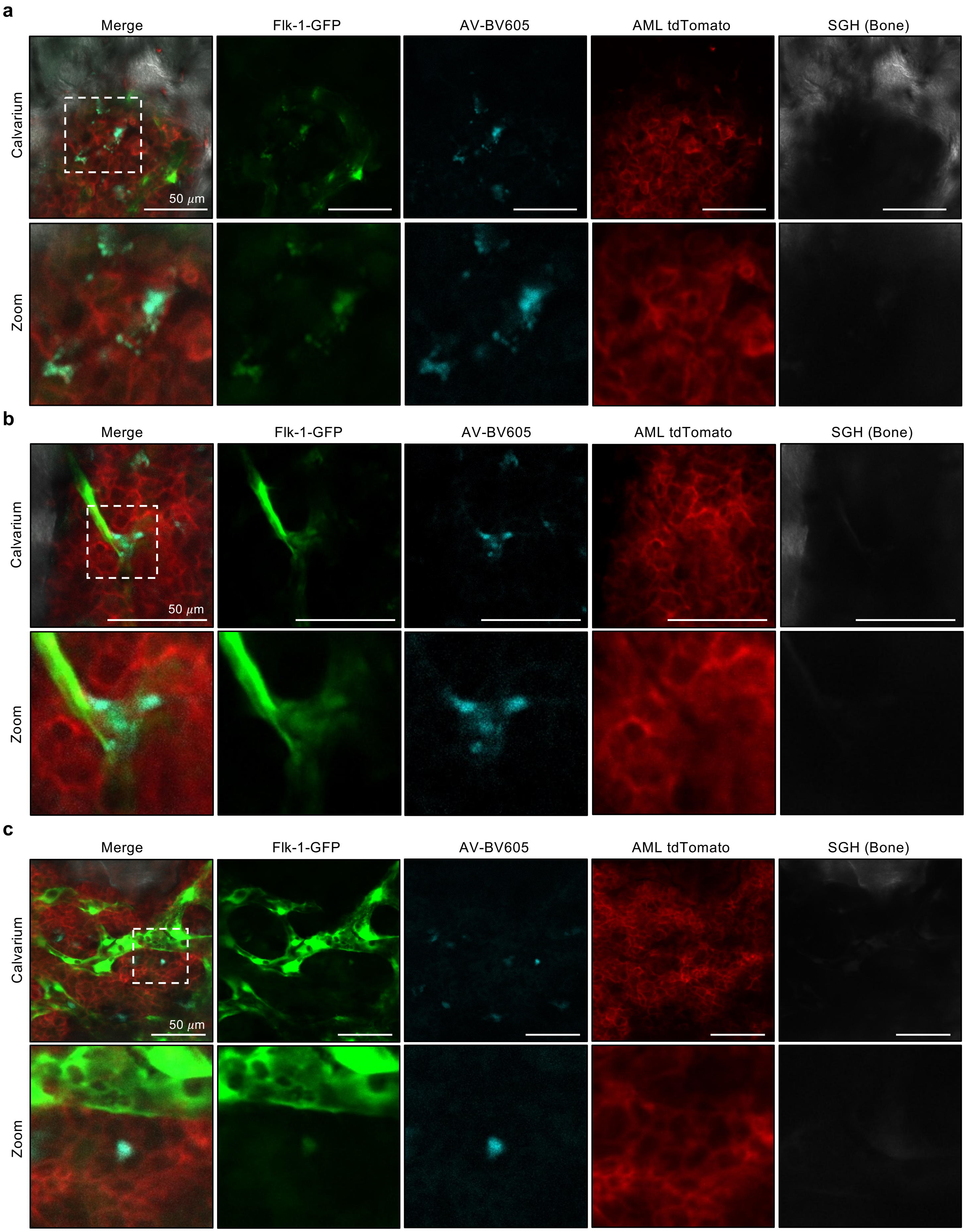

Supplementary Figure 9: AV$^+$ cells and fragments can be detected in the BM calvarium upon AML infiltration.
Steady state and AML-burdened mice (18-20 days post-transplantation with AML) were injected with ~100 μL fluorescently conjugated AV-BV605. AV$^+$ Flk1-GFP$^+$ endothelial cells and fragments could be detected in mice with AML infiltration by intravital microscopy of the BM calvarium (n=5).

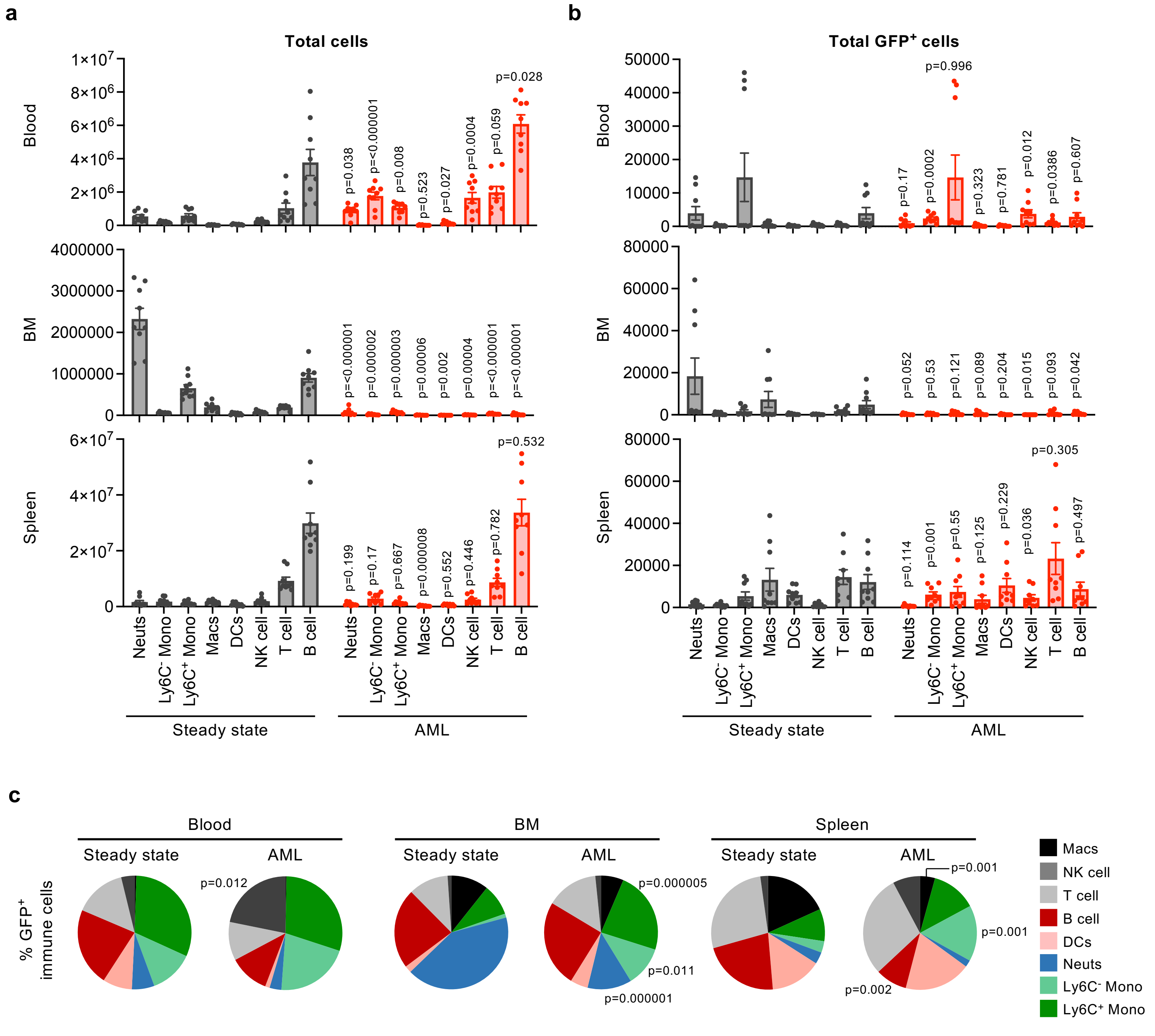

Supplementary Figure 10: Expansion of AML alters immune cell proportions in the blood, BM and spleen.
Flow cytometry immune cell profiles in steady state and AML-burdened mice showing the (**a**) absolute number, (**b**) total GFP^{+} and (**c**) percentage of GFP+ neutrophils, LyC6^{+} monocytes, Ly6C^{-} monocytes, macrophages, DCs, NK cells, T cells and B cells. P values not shown in c represent p = > 0.05. Statistical significance was determined by unpaired Students two tailed *t* test between steady state and AML, (n=9). Black = macrophages, dark grey = natural killer cells, light grey = T cells, dark red = B cells, light red = dendritic cells, blue = neutrophils, light green = Ly6C^{-} monocytes, dark green = Ly6C^{+} monocytes.
