# Supplementary material for: In situ visualization of endothelial cell-derived extracellular vesicle formation in steady state and malignant conditions
Source: Nat Commun. 2024 Oct 22;15:8802. doi: 10.1038/s41467-024-52867-5 (PMC11496675; doi:10.1038/s41467-024-52867-5)
Supplement: Supplementary file 3 — Description of Additional Supplementary Information [file 41467_2024_52867_MOESM3_ESM.pdf]

Description of Additional Supplementary Files

File Name: Supplementary Movie 1
Description: Steady State Flk1-GFP Calvarium. Intravital microscopy tile scan and z stack of a Flk1-GFP+ calvarium under steady state conditions. Green = Flk1-GFP; Grey = Bone.

File Name: Supplementary Movie 2
Description: In vivo timelapse of Flk1-GFP+ EV formation. Example of Flk1-GFP+ EV formation observed by intravital microscopy of the bone marrow calvarium. Data represents maximum intensity projection. Green = Flk1-GFP; Grey = Bone.

File Name: Supplementary Movie 3
Description: In vivo timelapse of Flk1-GFP+ EV formation. Example of Flk1-GFP+ EV formation observed by intravital microscopy of the bone marrow calvarium. Video represents a 3D processed version. Green = Flk1-GFP; Grey = Bone.

File Name: Supplementary Movie 4
Description: In vivo timelapse of Flk1-GFP+ EV formation. Example of Flk1-GFP+ EV formation observed by intravital microscopy of the bone marrow calvarium. Data presented as a 3D, rendered representation. Green = Flk1-GFP; Grey = Bone.

File Name: Supplementary Movie 5
Description: In vivo timelapse of Flk1-GFP+ EV formation. Example of Flk1-GFP+ EV formation observed by intravital microscopy of the bone marrow calvarium. Presented as a MIP. Green = Flk1-GFP. Scale bar = 20 μm.

File Name: Supplementary Movie 6
Description: In vivo timelapse of Flk1-GFP+ EV formation. Example of Flk1-GFP+ EV formation where apoptotic cell-like morphologies are observed by intravital microscopy of the bone marrow calvarium. Presented as a MIP. Green = Flk1-GFP. Scale bar = 20 μm.

File Name: Supplementary Movie 7
Description: Intravital microscopy of EV – endothelial cell interaction. Example of Flk1-GFP+ EV trafficking along the bone marrow vasculature in the calvarium and interacting with Flk1-GFP+ endothelial cells. Presented as a MIP. Green = Flk1-GFP.

File Name: Supplementary Movie 8
Description: Airyscan confocal microscopy of MTR+ Flk1- GFP+ EV. Confocal microscopy z stack of a MitoTracker Red+ (MTR) bone marrow-derived Flk1-GFP+ EV. Green = Flk1-GFP; magenta = MTR.

File Name: Supplementary Movie 9
Description: 4D analysis of Flk1-GFP+ endothelial cells. Example of 4D data acquired to assess cell division of Flk1-GFP+ endothelial cells in the bone marrow calvarium. Green = Flk1-GFP; Grey = Bone.

File Name: Supplementary Movie 10

Description: Time-lapse microscopy of kdrl-mCherry+ EVs. Time-lapse confocal microscopy of a zebrafish embryo showing kdrlmCherry+ blood vessels and EVs. Data presented as a maximum intensity projection and 3D render.

File Name: Supplementary Movie 11
Description: Annexin V+ Flk1-GFP+ cells and fragments in the BM calvarium of AML-burdened mice. Multiphoton z stack showing Annexin V+ Flk1-GFP+ cells and fragments in the bone marrow calvarium of AML-burdened mice, 18 days post-transplantation. Green = Flk1-GFP; Cyan = Annexin V-BV605; Red = MLL-AF9 tdTomato; Grey = Bone.

File Name: Supplementary Movie 12
Description: Steady State vs AML-burdened Flk1-GFP Calvarium. Multiphoton microscopy tile scan and z stack of the bone marrow calvarium under steady state conditions or at 18 days post-AML transplantation. Green = Flk1-GFP; Red = MLL-AF9 tdTomato; Grey = Bone.

File Name: Supplementary Movie 13
Description: In vivo time-lapse of Flk1-GFP+ EV formation during AML. Example of Flk1-GFP+ EV formation observed by time-lapse intravital microscopy of the bone marrow calvarium of an AMLburdened mouse, 18 days posttransplantation. Green = Flk1-GFP; Red = MLL-AF9 tdTomato; Grey = Bone.

File Name: Supplementary Movie 14
Description: In vivo time-lapse of Flk1-GFP+ EV formation and cell fragmentation during AML. Example of Flk1-GFP+ EV formation, cell fragmentation and lysis observed in the bone marrow calvarium of an AML-burdened mouse, 18 days post-transplantation, by intravital microscopy. Green = Flk1-GFP; Red = MLL-AF9 tdTomato; Grey = Bone.
