# Supplementary material for: In situ visualization of endothelial cell-derived extracellular vesicle formation in steady state and malignant conditions
Source: Nat Commun. 2024 Oct 22;15:8802. doi: 10.1038/s41467-024-52867-5 (PMC11496675; doi:10.1038/s41467-024-52867-5)
Supplement: Supplementary file 18 — Reporting Summary [file 41467_2024_52867_MOESM18_ESM.pdf]

nature portfolio

Corresponding author(s): Ivan Poon, Edwin Hawkins and Georgia Atkin-Smith

Last updated by author(s): Sep 12, 2024

Reporting Summary

Nature Portfolio wishes to improve the reproducibility of the work that we publish. This form provides structure for consistency and transparency in reporting. For further information on Nature Portfolio policies, see our Editorial Policies and the Editorial Policy Checklist.

Statistics

For all statistical analyses, confirm that the following items are present in the figure legend, table legend, main text, or Methods section.

n/a	Confirmed	
☐	☒	The exact sample size (*n*) for each experimental group/condition, given as a discrete number and unit of measurement
☐	☒	A statement on whether measurements were taken from distinct samples or whether the same sample was measured repeatedly
☐	☒	The statistical test(s) used AND whether they are one- or two-sided<br>*Only common tests should be described solely by name; describe more complex techniques in the Methods section.*
☒	☐	A description of all covariates tested
☒	☐	A description of any assumptions or corrections, such as tests of normality and adjustment for multiple comparisons
☐	☒	A full description of the statistical parameters including central tendency (e.g. means) or other basic estimates (e.g. regression coefficient) AND variation (e.g. standard deviation) or associated estimates of uncertainty (e.g. confidence intervals)
☐	☒	For null hypothesis testing, the test statistic (e.g. *F*, *t*, *r*) with confidence intervals, effect sizes, degrees of freedom and *P* value noted<br>*Give P values as exact values whenever suitable.*
☒	☐	For Bayesian analysis, information on the choice of priors and Markov chain Monte Carlo settings
☒	☐	For hierarchical and complex designs, identification of the appropriate level for tests and full reporting of outcomes
☒	☐	Estimates of effect sizes (e.g. Cohen's *d*, Pearson's *r*), indicating how they were calculated

Our web collection on statistics for biologists contains articles on many of the points above.

Software and code

Policy information about availability of computer code

Data collection	BD Cell Sorter Aria III, BD Cell Sorter Aria W, BD Fortessa X-20, BD FACS Canto II, Backman Coulter CytoFLEX, Amnis ImageStreamX MkII collected with INSPIRE Software, ACRF Olympus FVMPE-RS Multiphoton microscope, Olympus FVMPE-RS Multiphoton microscope, Zeiss LSM 880 NLO Dual Confocal Multiphoton microscope, Zeiss LSM 880 lasar scanning confocal microscope.
Data analysis	Imaging flow cytometry was analysed by IDEAS version 6 Software (Amnis). Flow cytometry and fluroscence activated cell sorting data was analysed by FlowJo software, version 10.8.2. Multiphoton and confocal microscopy data was analysed in Fiji (Image J) version 2.9.0 and Imaris (Bitplane Scientific Software). Raw data was visualised and processed using Microsoft Excel and GraphPad Prism, version 10.2.3.

For manuscripts utilizing custom algorithms or software that are central to the research but not yet described in published literature, software must be made available to editors and reviewers. We strongly encourage code deposition in a community repository (e.g. GitHub). See the Nature Portfolio guidelines for submitting code & software for further information.

Data

Policy information about availability of data

All manuscripts must include a data availability statement. This statement should provide the following information, where applicable:

- Accession codes, unique identifiers, or web links for publicly available datasets
- A description of any restrictions on data availability
- For clinical datasets or third party data, please ensure that the statement adheres to our policy

All data presented in this study is original and not published elsewhere. All data supporting the findings of this study are available within the paper, within the

supplementary information and within the Source Data File. Additional information is available upon request to i.poon@latrobe.edu.au, hawkins.e@wehi.edu.au or atkinsmith.g@wehi.edu.au.

Research involving human participants, their data, or biological material

Policy information about studies with human participants or human data. See also policy information about sex, gender (identity/presentation), and sexual orientation and race, ethnicity and racism.

Reporting on sex and gender	The study included both male (n=3; age 48-67; average age 58) and female (n=8; age 26-60; average age 44) volunteers.
Reporting on race, ethnicity, or other socially relevant groupings	Data not collected
Population characteristics	All volunteers self-identified as healthy, with no clinical diagnoses or any inflammatory disorders. Current medications were noted for each individual. Four participants reported using the following medications: Simicort inhaler, Ventolin, Vitamin D supplement, Pristiq.
Recruitment	Healthy donors were recruited through the Volunteer Blood Donor Registry (VBDR) at WEHI to obtain a broad range of ages and sexes across individuals with no known inflammatory. Donor age, sex, and medical history was self-reported.
Ethics oversight	Ethical approval for this study was granted by the WEHI Human Research Ethics Committee (project ID: 10/02). Written, informed consent was obtained from all participants prior to their participation, in accordance with the Declaration of Helsinki and its later amendments.

Note that full information on the approval of the study protocol must also be provided in the manuscript.

Field-specific reporting

Please select the one below that is the best fit for your research. If you are not sure, read the appropriate sections before making your selection.

- [x] Life sciences
- [ ] Behavioural & social sciences
- [ ] Ecological, evolutionary & environmental sciences

For a reference copy of the document with all sections, see nature.com/documents/nr-reporting-summary-flat.pdf

Life sciences study design

All studies must disclose on these points even when the disclosure is negative.

Sample size	Sample size calculations were not performed. Sample sizes for each experiment where chosen to be consistent with the field norms.
Data exclusions	Outliers were identified by performing a ROUT Outlier test in GraphPad Prism.
Replication	Unless otherwise specified, all experiments contain 3 independent repeats.
Randomization	NA
Blinding	Blinding occurred exclusively for quantification of caspase 3 staining on long bone sections and vessel density measurements.

Reporting for specific materials, systems and methods

We require information from authors about some types of materials, experimental systems and methods used in many studies. Here, indicate whether each material, system or method listed is relevant to your study. If you are not sure if a list item applies to your research, read the appropriate section before selecting a response.

Materials & experimental systems

n/a	Involved in the study	
[ ]	[x]	Antibodies
[ ]	[x]	Eukaryotic cell lines
[x]	[ ]	Palaeontology and archaeology
[ ]	[x]	Animals and other organisms
[x]	[ ]	Clinical data
[x]	[ ]	Dual use research of concern
[x]	[ ]	Plants

Methods

n/a	Involved in the study	
[x]	[ ]	ChIP-seq
[ ]	[x]	Flow cytometry
[x]	[ ]	MRI-based neuroimaging

Antibodies

Antibodies used

- CD45.2-PerCP Cy5.5 (BD Bioscience, #552950)
- CD45.2 AF700 (BD Bioscience, #560694)
- CD45.2-PE (BD Bioscience, #560695)
- CD45.2-APC Cy7 (BD Bioscience, #560694)
- CD11b-500 (BD Bioscience, #562127)
- CD11c-APC Cy7 (BD Bioscience, #561241)
- Ly6C-BV421 (BD Bioscience, #562727)
- Ly6G-PeCy7 (BD Bioscience, #560601)
- F4/80-PeCy5 (eBioscience, #15-4801-82)
- CD64-BV786 (BD Bioscience, #741024)
- I-A/I-E-BV650 (BD Bioscience, #743873)
- CD3-AF700 (BioLegend, #100215)
- CD4-BV421 (BD Bioscience, #562891)
- CD8-BV650 (BioLegend, #100742)
- CD19-PeCy7 (BD Bioscience, #552854)
- NK1.1-APC Cy7 (BD Bioscience, #560618)
- Flk1-PE (Invitrogen, #12-5821-82)
- CD31-PeCy7 (eBioscience, #25-0311-82)
- CD146-PerCP Cy5.5 (BD Bioscience, #562231)
- CD41-APC Cy7 (BioLegend, #133928)
- Tomm20-647 (Abcam, #ab209606)
- Flk1-PE (BD Bioscience, 560872)
- CD31-PeCy7 (BD Bioscience, 563651)
- CD144-BV786 (BD Bioscience, 565672)
- CD133-BV421 (BD Bioscience, 566595)
- CD45-V500 (BD Bioscience, 560777)
- Cleaved Caspase 3 (Cell Signalling Technology, 9661)
- Goat anti-Rabbit AF633 (ThermoFisher, A-21070)

Validation

- CD45.2-PerCP Cy5.5 (BD Bioscience, #552950): Validation stated on manufacturers' website (flow cytometry routinely tested)
- CD45.2 AF700 (BD Bioscience, #560694): Validation stated on manufacturers' website (flow cytometry routinely tested)
- CD45.2-PE (BD Bioscience, #560695): Validation stated on manufacturers' website (flow cytometry routinely tested)
- CD45.2-APC Cy7 (BD Bioscience, #560694): Validation stated on manufacturers' website (flow cytometry routinely tested)
- CD11b-500 (BD Bioscience, #562127): Validation stated on manufacturers' website (flow cytometry routinely tested)
- CD11c-APC Cy7 (BD Bioscience, #561241): Validation stated on manufacturers' website (flow cytometry routinely tested)
- Ly6C-BV421 (BD Bioscience, #562727): Validation stated on manufacturers' website (flow cytometry routinely tested)
- Ly6G-PeCy7 (BD Bioscience, #560601): Validation stated on manufacturers' website (flow cytometry routinely tested)
- CD64-BV786 (BD Bioscience, #741024): Validation stated on manufacturers' website (flow cytometry qualified)
- I-A/I-E-BV650 (BD Bioscience, #743873) : Validation stated on manufacturers' website (flow cytometry qualified)
- CD4-BV421 (BD Bioscience, #562891): Validation stated on manufacturers' website (flow cytometry routinely tested)
- CD19-PeCy7 (BD Bioscience, #552854): Validation stated on manufacturers' website (flow cytometry routinely tested)
- NK1.1-APC Cy7 (BD Bioscience, #560618): Validation stated on manufacturers' website (flow cytometry routinely tested)
- CD146-PerCP Cy5.5 (BD Bioscience, #562231): Validation stated on manufacturers' website (flow cytometry routinely tested)
- CD31-PeCy7 (BD Bioscience, 563651): Validation stated on manufacturers' website (flow cytometry routinely tested)
- CD144-BV786 (BD Bioscience, 565672): Validation stated on manufacturers' website (flow cytometry routinely tested)
- CD133-BV421 (BD Bioscience, 566595): Validation stated on manufacturers' website (flow cytometry routinely tested)
- CD45-V500 (BD Bioscience, 560777): Validation stated on manufacturers' website (flow cytometry routinely tested)
- Flk1-PE (BD Bioscience, 560872): Validation stated on manufacturers' website (flow cytometry routinely tested)
- F4/80-PeCy5 (eBioscience, #15-4801-82): Manufacturer states reported validation in flow cytometry analysis
- CD31-PeCy7 (eBioscience, #25-0311-82): Manufacturer states reported validation in flow cytometry analysis
- CD41-APC Cy7 (BioLegend, #133928): Validation stated on manufacturers' website (flow cytometry quality tested)
- CD3-AF700 (BioLegend, #100215): Validation stated on manufacturers' website (flow cytometry quality tested)
- CD8-BV650 (BioLegend, #100742): Validation stated on manufacturers' website (flow cytometry quality tested)
- Flk1-PE (Invitrogen, #12-5821-82): Manufacturer states reported validation in flow cytometry analysis
- Tomm20-647 (Abcam, #ab209606): Manufacturer states predicted reactivity for mouse immunofluorescence
- Cleaved Caspase 3 (Cell Signalling Technology, #9661): Validation stated on manufacturers' website for flow cytometry, immunofluorescence, immunohistochemistry and western blotting
- Goat anti-Rabbit AF633 (ThermoFisher, A-21070): Validation stated on manufacturers' website for flow cytometry, immunocytochemistry and immunohistochemistry

Eukaryotic cell lines

Policy information about cell lines and Sex and Gender in Research

Cell line source(s)

J774A.1 macrophages were acquired from ATCC.
EuMyc 560 cells are female (XX chromosome).

	X/Y chromosomes of MLL-AF9 and T-ALL primary cells were not determined by genetic screening. These primary cells lines were previously used and published in Duarte et al. 2018 Cell Stem Cell and Hawkins et al. 2016 Nature.
Authentication	Cell lines were not authenticated by genomic analysis but their response to stimuli and morphologies were consistent with their designated origins.
Mycoplasma contamination	Cell lines were not routinely tested for mycoplasma.
Commonly misidentified lines (See ICLAC register)	No commonly misidentified cell lines were used in this study.

Animals and other research organisms

Policy information about studies involving animals; ARRIVE guidelines recommended for reporting animal research, and Sex and Gender in Research

Laboratory animals	Vegf-r2 or Fetal liver kinase 1 (Flk1)-GFP mice (Flk1GFP/+) were bred and housed in specific pathogen-free (SPF) conditions at the WEHI animal facility (Parkville, VIC, Australia). Atg7fl/fl (Atg7tm1Tchi) mice were provided by the Kile Laboratory (University of Adelaide, SA, Australia). The UBC-CreERT2 mice were provided by the Heath Laboratory (WEHI, VIC, Australia). Mice were housed at the La Trobe Animal Research and Teaching Facility (LARTF, La Trobe University, VIC, Australia) under SPF conditions. To induce deletion in Atg7fl/fl UBC-CreERT2+/- mice, mice aged six weeks or older were intraperitoneally injected with 4 mg tamoxifen (Sigma-Aldrich, #T5648) in sunflower seed oil (Sigma-Aldrich, #25007), delivered as one injection per day of 200 mL of a 10 mg/mL stock, over two consecutive days. Targeted deletion of the Mertk gene in Cx3Cr1+ cells was achieved by crossing Mertk-floxed mice with mice expressing cre recombinase under the regulatory control of the endogenous Cx3Cr1 promoter (C57BL/6-Cx3cr1<tm1.1(cre) Jung>/Orl Cx3Cr1cre). MertK-flox mice were maintained at the Florey Institute of Neuroscience and Mental Health, VIC, Australia, in a SPF environment. All mice were maintained on a C57Bl/6 background.<br><br>Adult TU (Tübingen) wt and Tg(kdrl:mCherry) zebrafish were housed at the LARTF, La Trobe University and maintained at 28°C on a 12 h light/12 h dark cycle. Embryos were collected in E3 embryo medium (5mM NaCl, 0.17 mM KCl, 0.33 mM CaCl2, 0.33 mM MgSO4) and treated with 0.003% (w/v) 1-phenyl-2-thiourea (PTU; Sigma, #P7629) from 24 h post-fertilization to inhibit pigmentation.
Wild animals	NA
Reporting on sex	Both male and female animals were used for all experiments and therefore sex-based differences were not assessed.
Field-collected samples	NA
Ethics oversight	All mouse experiments were performed in accordance with the WEHI animal ethics committee regulations (AEC 2021.007) and the La Trobe University animal ethics committee (AECs 18024, 18036) in accordance with the Australian code for the care and use of animals for scientific purposes.<br><br>All zebrafish work was conducted under AEC19002 as approved by La Trobe University Animal Ethics Committee and in accordance with the Australian code for the care and use of animals for scientific purposes.

Note that full information on the approval of the study protocol must also be provided in the manuscript.

Plants

Seed stocks	*Report on the source of all seed stocks or other plant material used. If applicable, state the seed stock centre and catalogue number. If plant specimens were collected from the field, describe the collection location, date and sampling procedures.*
Novel plant genotypes	*Describe the methods by which all novel plant genotypes were produced. This includes those generated by transgenic approaches, gene editing, chemical/radiation-based mutagenesis and hybridization. For transgenic lines, describe the transformation method, the number of independent lines analyzed and the generation upon which experiments were performed. For gene-edited lines, describe the editor used, the endogenous sequence targeted for editing, the targeting guide RNA sequence (if applicable) and how the editor was applied.*
Authentication	*Describe any authentication procedures for each seed stock used or novel genotype generated. Describe any experiments used to assess the effect of a mutation and, where applicable, how potential secondary effects (e.g. second site T-DNA insertions, mosiacism, off-target gene editing) were examined.*

Flow Cytometry

Plots

Confirm that:

- [x] The axis labels state the marker and fluorochrome used (e.g. CD4-FITC).
- [x] The axis scales are clearly visible. Include numbers along axes only for bottom left plot of group (a 'group' is an analysis of identical markers).
- [x] All plots are contour plots with outliers or pseudocolor plots.
- [x] A numerical value for number of cells or percentage (with statistics) is provided.

Methodology

Sample preparation	For murine tissue samples:<br>Blood was harvested in EDTA K microtubes and immediately lysed in red blood cell (RBC) lysis buffer for 5 min at room temperature (RT). Samples were topped with 1 mL PBS and centrifuged at 3000 g for 6 min before repeating RBC lysis. The BM samples were prepared by flushing tibias/femurs/hips with PBS using a 1 mL syringe and 25-gauge needle. Samples were filtered through a 70 um cell strainer and centrifuged at 3000 g for 6 min. For spleen analysis, spleens were harvested and filtered directly through a 70 um cell strainer into 5 mL of RBC lysis buffer and incubated for 5 min at RT. Samples were topped with PBS and centrifuged at 3000 g for 6 min.<br><br>For fluorescence Activated Cell Sorting (FACS) of murine samples:<br>To isolate BM-derived EVs via FACS, EVs were first enriched by differential centrifugation. Briefly, multiple flushed BM samples were pooled and centrifuged at 300 g for 10 min to pellet cells. The supernatant was collected and centrifuged at 3000 g for 20 min to pellet EVs. Samples were resuspended in FACS buffer (10% (v/v) FCS, 2 mM EDTA, PBS, 1x Annexin V (AV) binding buffer), stained and filtered through a 70 um cell strainer prior to sorting. To isolate GFP+ immune cells for confocal microscopy, BM samples were processed as above however, the 300 g cell pellet was used for subsequent staining and sorting.<br><br>For human peripheral blood analysis:<br>Whole blood was diluted at 1:1 with PBS and separated by Ficoll gradient. The plasma and mononuclear cell fractions were harvested and centrifuged at 300 g for 10 min to pellet cells. The supernatant was collected and centrifuged at 3000 g for 10 min to pellet EVs.<br><br>For zebrafish embryo analysis:<br>5-10 embryos were pooled together and chilled on ice. Embryos were rinsed in sterile calcium-free Ringer's solution with 2.5 mM EDTA for 15 min on a roller at 4°C. Samples were gently resuspended to deyolk embryos and rinse solution was replaced with 0.25% (w/v) trypsin/EDTA for 15 min at 28'C. Samples were resuspended to disassociate embryos and 1x PBS / 1% (w/v) BSA was added to inactivate trypsin. Single cell solutions were filtered through a 40 um cell strainer and centrifuged at 3000 g for 5 min to pellet cells and EVs.
Instrument	BD Fortessa X-20, BD FACS Canto II, Backman Coulter CytoFLEX
Software	The software used to collect and analyse flow cytometry data was BD FACSDiva 8.0.1 and FlowJo 10.8.2.<br>CytExpert Software version 2.5 was used to collect data on the Beckman Coulter CytoFlex.
Cell population abundance	For flow cytometry analysis, between 0.2-1x10^6 events were typically acquired. Purity of EVs and GFP+ immune cells isolated by FACS were confirmed by post-FACS confocal microscopy.
Gating strategy	Frequently used gating strategies underpinning novel analysis in the manuscript are located in the Supplementary Figures. Further details and additional gating strategies are available upon request to i.poon@latrobe.edu.au, hawkins.e@wehi.edu.au or atkinsmith.g@wehi.edu.au.

- [x] Tick this box to confirm that a figure exemplifying the gating strategy is provided in the Supplementary Information.
